# Human thioredoxin, a damage-associated molecular pattern and *Malassezia*-crossreactive autoallergen, modulates immune responses via the C-type lectin receptors Dectin-1 and Dectin-2

**DOI:** 10.1038/s41598-019-47769-2

**Published:** 2019-08-01

**Authors:** L. M. Roesner, M. Ernst, W. Chen, G. Begemann, P. Kienlin, M. K. Raulf, B. Lepenies, T. Werfel

**Affiliations:** 1 Hannover Medical School, Department of Dermatology and Allergy, Division of Immunodermatology and Allergy Research, Hannover, Germany; 2 University of Veterinary Medicine Hannover, Immunology Unit & Research Center for Emerging Infections and Zoonoses (RIZ), Hannover, Germany; 3 University of Veterinary Medicine Hannover, Institute for Parasitology, Hannover, Germany

**Keywords:** Cytokines, Inflammation, Medical research

## Abstract

Human thioredoxin (hTrx), which can be secreted from cells upon stress, functions in allergic skin inflammation as a T cell antigen due to homology and cross-reactivity with the fungal allergen Mala s13 of the skin-colonizing yeast *Malassezia sympodialis*. Recent studies have shown that cell wall polysaccharides of *Malassezia* are detected by the immune system via the C-type lectin receptors Dectin-1 and Dectin-2, which are expressed on myeloid cells. Therefore, this study aimed to investigate a putative interaction between Dectin-1, Dectin-2 and the allergens Mala s13 and hTrx. Stimulation of human monocyte-derived dendritic cells or macrophages with Mala s13 or hTrx resulted in remarkable secretion of IL-1β and IL-23. Blocking experiments suggest that hTrx induces IL-23 by Dectin-1 binding and IL-1β by binding to either Dectin-1 or Dectin-2. Regarding Mala s13, Dectin-1 appears to be involved in IL-1β signaling. Interference of Syk kinase function was performed to investigate downstream signaling, which led to diminished hTrx responses. In our experiments, we observed rapid internalization of Mala s13 and hTrx upon cell contact and we were able to confirm direct interaction with Dectin-1 as well as Dectin-2 applying a fusion protein screening platform. We hypothesize that this cytokine response may result in a Th2/Th17-polarizing milieu, which may play a key role during the allergic sensitization in the skin, where allergen presentation to T cells is accompanied by microbial colonization and skin inflammation.

## Introduction

Autoimmunity and allergy represent two examples of an aberrant reaction of the immune system, where immune responses are mounted against harmless self or environmental antigens. A critical step during the sensitization process seems to be the generation of memory T and B cells. While allergen- and autoantigen-specific T cells are tolerogenic in healthy donors, the immune system of sensitized individuals considers allergens or autoantigens to be dangerous. The sensitization process is thought to result from a situation, where antigen recognition takes place under parallel heavy attack of the immune system. It is believed that danger signals are present under such circumstances, which influence the antigen presenting cells (APC) towards a pro-inflammatory phenotype, leading to the generation of specific pro-inflammatory T cells. This break of tolerance is regarded as a key point in the development of autoimmunity and allergy. While many molecules have been described to act as a danger signal, their potential to shape the immune system is often unclear.

Using tonsillar cells of allergic patients, it was shown that stimulation of APC via toll-like receptors (TLR) 4 and 8 as well as the cytokines IL-1β and IL-6 have the potential to break specific T cell tolerance towards several environmental allergens^1^. A similar mechanism is discussed for DAMPs (danger-associated molecular pattern) and PAMPs (pathogen-associated molecular pattern)^2,3^.

A disease setting, where allergy, DAMPs and PAMPs are in a close interaction, is atopic dermatitis (AD), the most common inflammatory skin disease worldwide^4^. Being our outermost barrier, the skin is colonized with a vast amount of microorganisms of which *Staphylococcus aureus* is the most widely studied. AD patients are also frequently colonized with and IgE-sensitized against the opportunistic yeast *Malassezia sympodialis*^5^. Antifungal treatment leads to an improvment of skin symptoms^6^, which may be due to the extinction of fungal allergens but also due reduced PAMP burden. Recent studies have shown that *Malassezia* PAMPs are recognized by Dectin-1 and Dectin-2, members of the pattern recognition receptor (PRR) family of C-type lectin receptors (CTLR), leading to NLRP3 inflammasome activation and finally IL-1β production^7,8^. CTLR are well-known for sensing fungal cell wall glucans or mannans, but also non-sugar molecules, such as DAMPs, have been described as ligands^9–11^. Generally, DAMPs like chaperones, heat-shock proteins and stress proteins have been linked to autoimmune disorders^12^, suggesting an intrinsic mechanism.

Interestingly, we and others showed in the past, that a genetically highly conserved human paralogue of the *Malassezia* allergen Mala s13, namely thioredoxin (hTrx) also acts as a DAMP on the immune system and is released upon stress from skin cells^13–16^. In detail, hTrx leads to the release of IL-13 and IL-10 from human peripheral blood mononuclear cells, while the IL-10 response is strikingly diminished in IgE-sensitized patients^14^.

To investigate whether *Malassezia* PAMPs and related human DAMPs may be ligands for CTLR, we performed specific binding and blocking experiments on monocyte-derived dendritic cells and macrophages as model APCs *in vitro*.

Finally, we show that Mala s13 and hTrx, but not Mala s6 or Mala s11, can be bound by Dectin-1 and Dectin-2. Blocking experiments suggest however that the IL-23 response to hTrx is mediated partially by Dectin-1, and the IL-1β response by Dectin-1 and -2. Regarding Mala s13, Dectin-1 appears to be involved in IL-1β signaling. Such pro-inflammatory capacities have been reported to facilitate sensitization in atopic individuals.

## Results

### Cytokine response of myeloid cells to fungal glucans, the fungal allergen Mala s13 and its human paralogue hTrx

**Figure 1:**
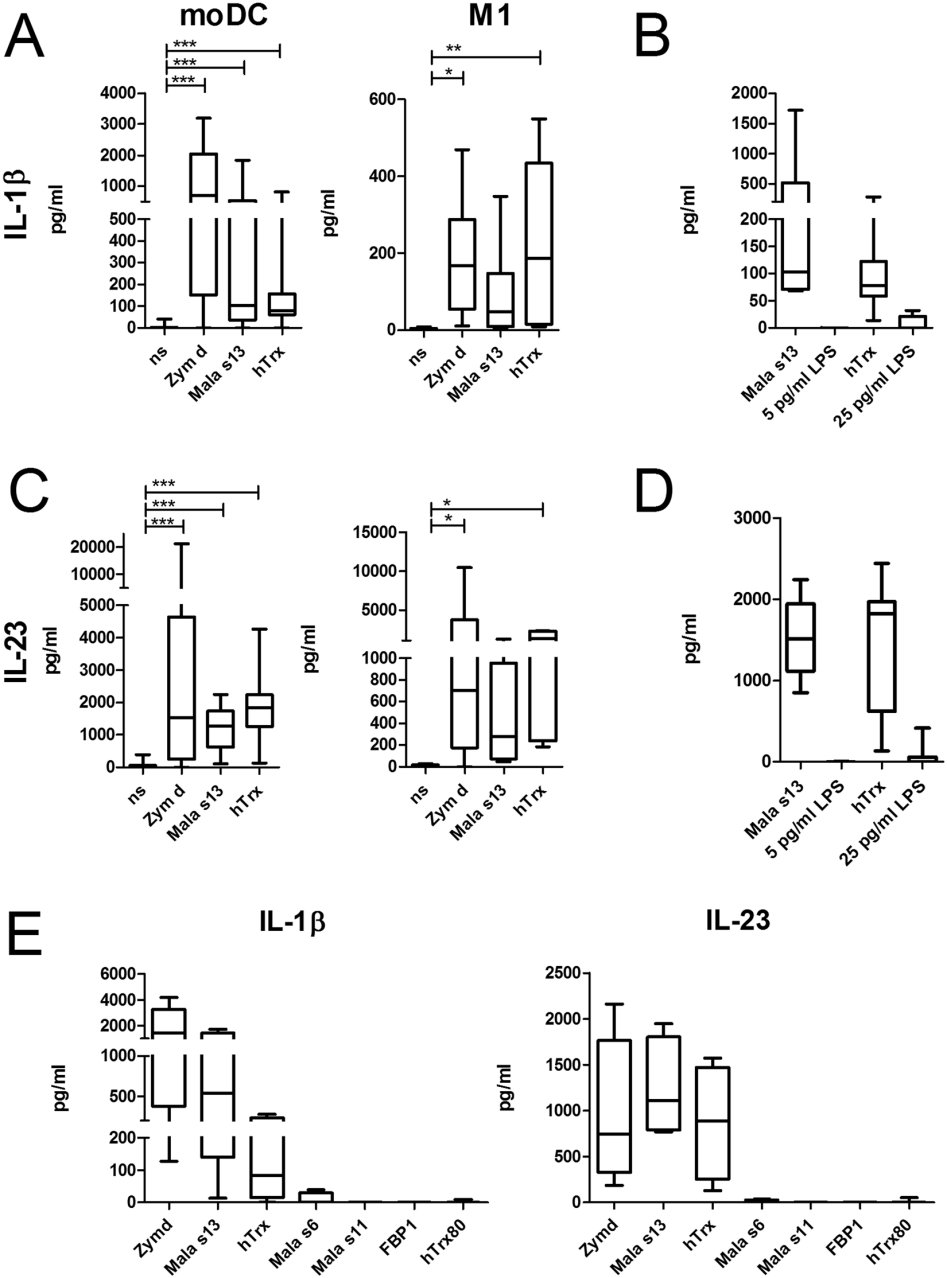
Cytokine response to fungal glycans, the fungal allergen Mala s13, and its human homologue hTrx. Secretion of IL-1β (**A**) and IL-23 (**C**) was measured in moDC or M1-macrophage cell culture supernatants by ELISA 16–18 h after stimulation as depicted (moDC n = 25; M1-macrophages n = 6). IL-1β (**B**) and IL-23 (**D**) release by moDC in response to the Mala s13, hTrx and the respective LPS contamination levels in the assay, 5 pg/ml and 25 pg/ml (n = 7). (**E**) Cytokine response to Zymosan depleted, Mala s13, and hTrx compared to further recombinant microbial antigens (Mala s6, Mala s11, FBP1) and a truncated form of hTrx (Trx80) (n = 6).

Monocyte-derived dendritic cells (moDC) and M1-macrophages have been shown to express the CTLR Dectin-1 and Dectin-2 and are well-studied regarding the binding of fungal glucans via the Dectin-1 receptor. In order to investigate their response to Mala s13 and hTrx, cytokine secretion was measured by ELISA after 16 h stimulation. Both cell types released considerable amounts of IL-1β and IL-23 in response to both proteins as well as to hot alkali-treated Zymosan (Zymosan depleted), a *S*. *cerevisae*-cell wall preparation that is described as a specific ligand of Dectin-1 but not of TLR (Fig. 1A,C). Remaining lipopolysaccharide (LPS)-contamination of recombinantly *E*. *coli*-expressed Mala s13 and hTrx was determined by LAL-assay. Equivalent LPS concentrations did not lead to IL-1β secretion in our assay (Fig. 1B,D); cytokine secretion in response to LPS was detectable in our system only upon a concentration of 2 ng/ml or higher (Supplemental Figure S1).

To exclude an artefact generated by recombinantly expressed polyhistidine-tagged proteins *per se*, moDCs were stimulated with the *Malassezia* allergens Mala s6 and Mala s11 and *S*. *aureus* fibronectin-binding protein1 (FBP1) which were expressed and purified equivalently. Further, we tested whether a cleavage product of hTrx, Trx80, is sufficient for the observed cytokine release. However, neither IL-1β nor IL-23 was released by any of these antigens, suggesting a specific receptor (Fig. 1E).

### Blocking of Dectin-1 decreases cytokine response

**Figure 2:**
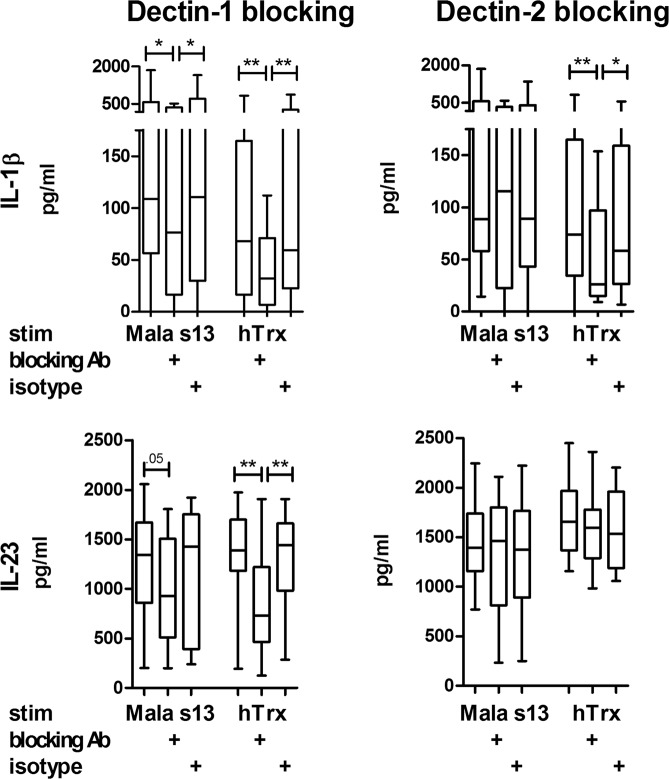
Blocking of Dectin decreases cytokine response to hTrx. Secretion of IL-1β and IL-23 was measured in moDC cell culture supernatants by ELISA 16–18 h after stimulation with Zymosan depleted, Mala s13, and hTrx with or without preliminary 1 h incubation with blocking antibodies against Dectin-1 or Dectin-2 as indicated (n = 12). Blocking antibodies were controlled applying isotype controls.

To test whether Dectin-1 and/or Dectin-2 recognize Mala s13 and hTrx, receptor blocking applying specific antibodies was performed prior to stimulation. Blocking Dectin-1 on moDC had a clear inhibitory effect on IL-1β and IL-23 cytokine release upon hTrx stimulation. IL-1β responses to Mala s13 were also reduced significantly in our set of experiments. The blockade of Dectin-2 also impaired the IL-1β release in response to hTrx, which was significantly different from the isotype control, but not IL-23 and not the response to Mala s13 (Fig. 2).

**Figure 3:**
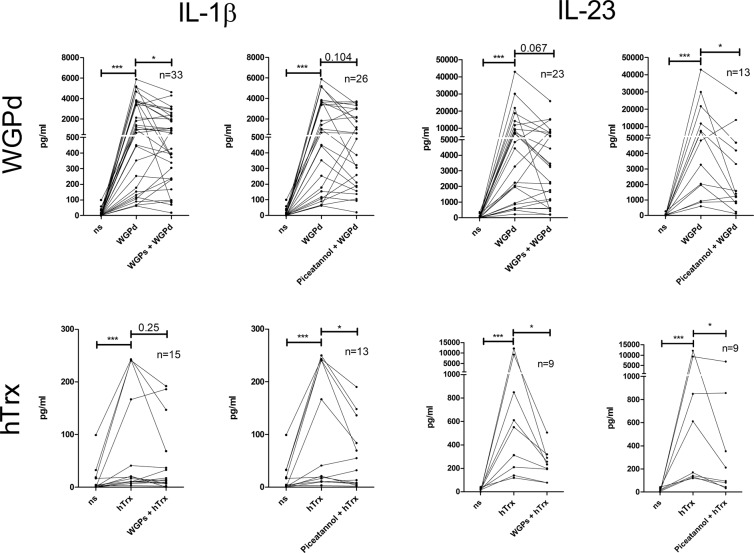
Blocking of Dectin-1 or Syk kinase decreases cytokine response to hTrx. Secretion of IL-1β and IL-23 was measured in moDC cell culture supernatants by ELISA 16–18 h after *Saccharomyces cerevisae* dispersable whole glucan particles (WGPd) and hTrx stimulation with or without preliminary 1 h incubation with *S*. *cerevisae* soluble whole glucan particles (WGPs) or piceatannol.

Particulate cell wall components of *Saccharomyces cerevisiae* (dispersable whole glucan particles, WGPd) have also been shown to specifically signal via Dectin-1^17^, while the soluble form (WGPs) is supposed to efficiently block this process^18^. In our set of experiments, stimulation with WGPd led to secretion of IL-23 and IL-1β, which was at least partially blocked by WGPs (Fig. 3). To confirm the results from Dectin-1-blocking antibodies above, hTrx was applied with and without pre-incubation with WGPs (Fig. 3). While WGPs was able to reduce the IL-23 secretion significantly, the IL-1β response was reduced only by trend in this set of experiments.

### Blocking of Syk kinase decreases cytokine response

CTLR that possess ITAM-motifs like Dectin-1 and Dectin-2 are well-known to signal intracellularly via the Syk kinase leading to the induction of cytokines and inflammasome activation^19^. Effects of WGPd have been shown to be efficiently diminished upon Syk blockade by the phenolic stilbenoid piceatannol. To investigate whether hTrx signaling follows this pathway, too, we blocked Syk kinase by piceatannol upfront hTrx stimulation, and found in our set of experiments that IL-23 and IL-1β secretion were reduced significantly (Fig. 3).

### Direct binding of Mala s13 and hTrx to Dectin-1 and Dectin-2

**Figure 4:**
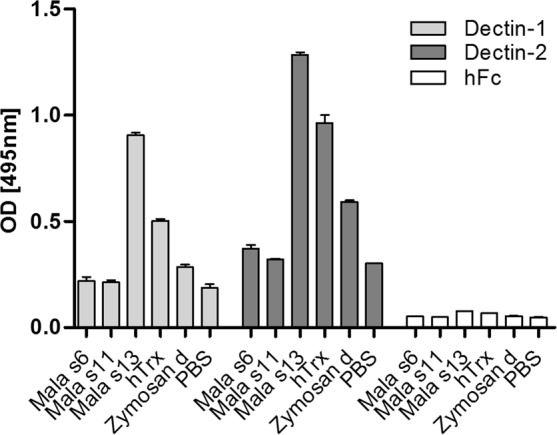
Binding of putative ligands to Dectin-1 and Dectin-2 was assessed applying a Dectin-hFc fusion protein screening platform. Bound fusion protein was assessed by an anti-hFC-antibody as optical density (OD). As a negative control, binding of each putative ligand to hFc only was tested (hFc). n = 2. All measurements were performed as duplicates. hTrx, human thioredoxin; PBS, phosphate buffered saline; Zymosan d, Zymosan depleted. Error bars represent SEM.

In order to clarify whether the interaction of Mala s13/hTrx and Dectin-1 is direct or indirect, binding strength to recombinant Dectin-1- and Dectin-2-Fc chain fusion molecules was determined according to an established protocol^20^. In this system, the extracellular part of Dectin is fused with the Fc-fragment of human IgG_1_, which can be detected by a HRP-labeled secondary antibody. As a control, binding of each test antigen was also determined to the empty hFc-fragment. Specific binding to Dectin-1 and Dectin-2 was clearly detectable for Mala s13 and hTrx as well as Zymosan, but not for Mala s6 and Mala s11 (Fig. 4). All recombinant proteins were intact and were applied in equal amounts, as verified by western blotting (Figure S2).

### Internalization of Dectin-1 and antigens after stimulation

**Figure 5:**
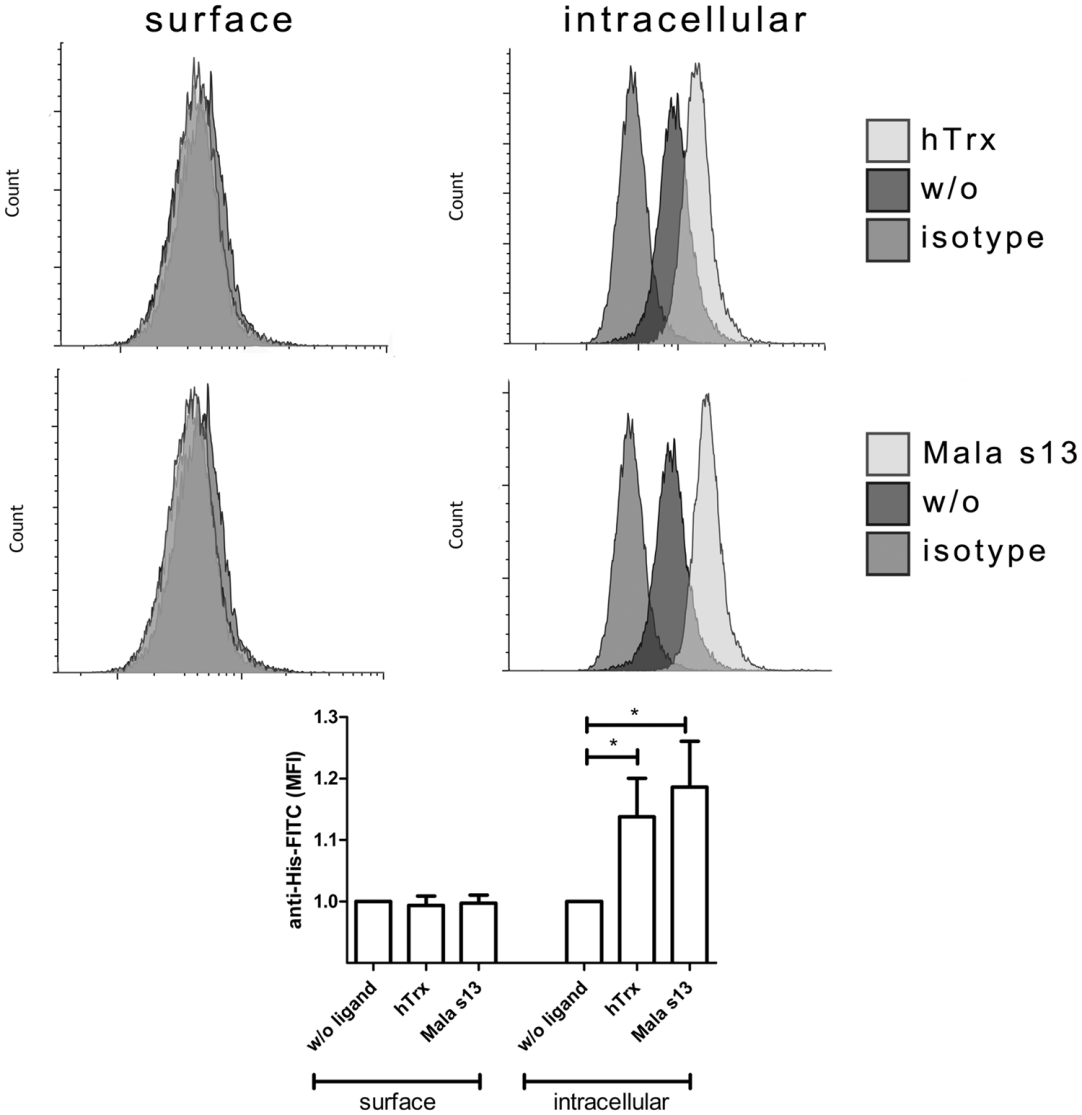
Surface and intracellular staining of Dectin-1 ligands. Recombinant Mala s13 and hTrx were detected inside moDC 10–20 min after incubation by a fluorescently labelled anti-6xHistidine-tag antibody. Prior to that, surface staining applying the same antibody was performed to block surface-bound antigens. Exemplary stainings are shown as indicated. w/o: medium control. Plotted graph shows arithmetic mean and SEM of six independent experiments, medium control is set to 1.

It has been reported that Dectin-1 and its ligands get internalized quickly upon binding^21^. To test if Mala s13 and hTrx follow this behavior, the internalization of the CTLR-antigen complex was investigated. After blocking to prevent unspecific binding, moDC were stimulated for 20 minutes with Mala s13 or hTrx. Since both antigens are 6xHis-tagged, detection was performed by immunofluorescence directed against the tag. Cells were stained on the cell surface first and subsequently intracellularly. The depicted increase in staining intensity when comparing surface to intracellular signals suggests the absence of both proteins on the cell surface but presence inside the cell (Fig. 5).

## Discussion

In this study we demonstrate in an *in vitro* model that human APC are capable of mounting a pro-inflammatory response to the fungal allergen Mala s13 as well as to its human highly-conserved paralogue, hTrx, via signaling in part through the CTLR Dectin-1 and Dectin-2. We can show strong IL-23 and IL-1β production in dendritic cells as well as macrophages, which both play important roles in the initiation and perpetuation of the skin inflammation in AD^4^. We show for the first time direct interaction of Mala s13 and hTrx to Dectin-1 and Dectin-2 on an established binding test platform^22^.

Crossreactivity between microbial and human antigens has also been described for *Aspergillus fumigatus*, also in the context of AD^23,24^. Interestingly, a recent study revealed upregulation of Dectin-1 and other pattern recognition receptors in patients suffering from aspergillosis^25^.

*Malassezia* species are part of a healthy skin microbiota and were recently identified as a target for Dectin-1, leading to the activation of the NLRP3 inflammasome and secretion of IL-1β^8^. Further, it has been demonstrated that Dectin-2 can also bind specifically to a *Malassezia* compound^7,26^. Since in a subgroup of AD patients Malassezia-specific IgE and T cell reactivities are observed, we hypothesize that the pro-inflammatory responses evoked via CTLR may underlie this phenomenon.

Dectin-1 has also been shown to have implications for allergic conditions, since mouse models of allergic asthma to house dust mite (HDM) showed reduced symptoms if Dectin-1 was knocked out^27,28^. It could be observed that parallel β-glucan signaling leads to exacerbation of allergy symptoms with a specific Th2/Th17 polarization phenotype. It could specifically be demonstrated that β-glucan can induce IL-17A directly and is furthermore able to enhance the HDM-induced secretion of IL-4 and IL-13 in a more than two-fold magnitude, resulting in a steroid-resistant phenotype of the disease^28^. Subsets of dendritic cells, as well as macrophages were identified as target cell populations^27,29^.

Within the CTLR family, Dectin-2 has been described to induce a Th2/Th17 allergic response to HDM, too, but rather by direct binding to HDM compounds^26,30–32^. In a previous study, we were able to confirm the presence of the Th2/17 HDM-reactive T cell subtype in patients suffering from AD^33^. This Th2/Th17 phenotype is in line with the cytokines detected here. While IL-23 is obligatory for survival of Th17 cells^34^, IL-1β has been reported as an important factor of the Th2 immune response^35^. In the context of allergy, Dectin-signaling may therefore promote the present type 2-response while fueling the fungus-associated Th17 axis in parallel.

CTLR are well-known for binding sugar residues and binding of Dectin-1 to β-glucan and Dectin-2 to α-mannans has been studied extensively. Therefore our findings upon stimulation with proteins may appear surprising. However, it has been demonstrated that proteins can also be for CTLR family members. For example, CLEC9A has been shown to bind F-actin, which under healthy circumstances resides inside the cell but gets exposed after cell death^36^. CLEC8A (also known as LOX-1 or SCARE-1) is a receptor for heat shock protein 70 (HSP70), which is a chaperone and well-described secreted alarmin^37^. Interestingly, CLEC8A-related proteins like Siglec, SREC-1 (SCARF-1) and FEEL-1 (SCARH-2) have also been reported to bind HSP-70. These three transmembrane receptors share homologous structures and react to low density lipoprotein (LDL) as a common ligand. Recently, Siglec-5 and Siglec-14 of the I-type lectin family have been shown to bind HSP70, too^10^. These examples may lead to the hypothesis that CTLR act as receptors for DAMPs and alarmins in the context of inflammation. This theory may explain the observed binding to hTrx here, since it is secreted upon stress^16^ and possesses distinct properties of a DAMP: we could show that healthy individuals mount a pronounced IFN-γ response accompanied by IL-10 from monocytes, while AD patients with detectable IgE-sensitization against hTrx elicit predominantly IL-13 with reduced IL-10 levels^14^.

It might be of interest to investigate whether the immune response described here can further be amplified via parallel signaling through other PRRs, like TLR2, TLR3, or TLR4^38,39^. Synergistic effects have also been reported upon parallel binding of fungal components by two different CTLRs^40^. On the other hand, some CTLR may also counteract others^41^.

Our findings of an interaction of a fungal allergen/PAMP and its human paralogous DAMP with Dectin-1 and Dectin-2 may help to understand the Th2/Th17 phenotype discovered in AD patients^33,42^. Therefore, our data underlines the rationale for a Th17-specific therapeutical intervention. The IL-17A-targeting biological Secukinumab which is approved in the treatment of psoriasis is already under investigation in AD (NCT02594098 and NCT03568136). Further, a human anti-IL-17C antibody is currently under study (NCT02739009). IL-23 can be targeted by Ustekinumab, an anti-IL-12/IL-23 antibody directly, and is under investigation in an interventional study with AD patients (NCT01806662).

Aside from AD, further roles of hTrx and Mala s13 can be speculated for other dermatological diseases, since it was shown that keratinocytes may express Dectin-1 on their surface after stimulation with β-glucan, resulting in an immune response^43^. This may be crucial for patients with AD, since the allergic inflammation and the site of *Malassezia* colonization co-localize in this disease setting.

## Material and Methods

ELISA: Cell culture supernatants were collected at indicated time points and cytokine secretion was measured by ELISA. IL-1β was determined by ELISA kit from R&D systems (Minneapolis, MN, USA) and IL-23 from affymetrix eBioscience (Santa Clara, CA, USA). Assays were conducted according to manufacturers’ instructions.

Allergens and stimuli: Recombinant Mala s6, s11, s13, and hTrx were produced as [His]_6_-tagged fusion proteins in *Escherichia coli* and purified by means of Ni^2+^ affinity chromatography (Qiagen, Hilden, Germany), as described previously^44^. FBP was produced by the same method, described by^45^. LPS contamination was determined by LAL test (Limulus Amebocyte Lysate, Pyrochrome, East Falmouth, MA, USA). The LPS concentration of hTrx was 10 ng/mg protein, corresponding to 25 pg/ml in the working concentration; the LPS content of Mala s13 was 1.5 ng/mg protein, corresponding to 3.75 pg/ml in the working concentration. Further, hTrx80 (Biotechne, Minneapolis, MN, USA) and Zymosan depleted (InvivoGen, San Diego, CA, USA) were included in stimulation assays.

Cell Culture: CD14-positive monocytes were isolated from buffy coats obtained from the local blood bank as well as volunteers. According to the guidelines for blood donation, the anonymous donors were healthy and had not taken any medication for four weeks before giving blood. No volunteer was under systemic immunosuppressive treatment and all blood donors provided their informed written consent. The study was conducted according to the Declaration of Helsinki Protocols and approved by the ethics committee of the Hannover Medical School (MHH). Monocytes were isolated to generate monocyte-derived human dendritic cells (MoDCs) or classical macrophages (M1). More precise, peripheral blood mononuclear cells (PBMCs) were isolated by density gradient centrifugation (Pancoll human, Pan Biotech, Aidenbach, Germany). Out of these, monocytes were isolated by CD14 labelled magnetic beads (MACS Miltenyi Biotech, Bergisch-Gladbach, Germany) following manufacturer’s instructions. Monocytes were cultured in RPMI (Biochrom, Berlin, Germany) supplemented with 5% FCS (Pan Biotech), 1 M Hepes (Biochrom), 2mM L-glutamine (Biochrom), 1% Penicillin/Streptomycin (Biochrom), 10 ng/ml recombinant human IL-4 (Biotechne) and 10 ng/ml recombinant human GM-CSF (Biotechne) for DCs for 7 days. In order to generate macrophages, the protocol was performed equivalently except the addition of IL-4. In blocking-experiments, DCs and M1 were incubated with m-anti-hDectin1- or m-anti-hDectin2-blocking antibodies (10 µg/ml, InvivoGen), whole glucan particles, soluble (WGPs, 1 µg/ml, InvivoGen), or 15 µg/ml piceatannol (Merck, Darmstadt, Germany) for 1 h and compared to isotype mIgG1 (10 µg/ml, BioLegend, San Diego, CA, USA) and mIgG2a (10 µg/ml, R&D), respectively. After incubation, cells were left unstimulated or were stimulated with 100 µg/ml Zymosan depleted (InvivoGen), 20 µg/ml whole glucan particles, dispersable (WGPd, InvivoGen), Mala s6, s11, s13, FBP1, hTrx80 (R&D), hTrx (all 2.5 µg/ml) or LPS as indicated (Sigma-Aldrich, Munich, Germany) for 16–18 h.

Flow cytometry: MoDCs were incubated with PE-labelled m-anti-hDectin-1 antibody (R&D, clone 559931) or respective mIgG2b isotype (BioLegend) for 45 minutes at 4 °C. Cells were washed three times with PBS (Pan Biotech) and analyzed on FACSCanto II (BD, Franklin Lakes, NJ, USA). 20.000 events were collected in each measurement.

Internalization: In order to detect internalization of putative Dectin-1 ligands, cell surface and intracellular immune staining was performed. moDCs were generated and stimulated as described above. 20 minutes post stimulation cells were fixed using 4% formaldehyde in PBS for 30 minutes at 4 °C. After blocking of unspecific binding, cells were stained by FITC-labelled mouse anti human 6xHistidine-epitope-tag antibodies (Acris, clone AD1.1.10) or respective isotype controls (mIgG1-FITC, Sigma-Aldrich) for 30 min on ice in PBS/0.2% gelatin. Subsequently, cells were either left untreated or treated with permeabilization buffer according to manufacturer´s instructions (Affymetrix ebioscience). A subsequent second round of staining was performed on all cells as described above in PBS/0.2% gelatin or permeabilization buffer, respectively. Cells were washed twice with PBS (Pan Biotech) or permeablilization buffer, respectively, and analyzed by flow cytometry on a FACSCanto II (BD).

Dectin-Fc binding assay: Dectin-1-Fc and Dectin-2-Fc fusion proteins were generated as described previously^20,46^. The sequence encoding the extracellular domain of Dectin receptors were ligated into the expression vector pFuse-hIgG1-Fc (InvivoGen, San Diego, CA) and transiently transfected into CHO-S cells using the FreeStyle Max CHO-S Expression System (Life Technologies, Darmstadt, Germany). Supernatant containing soluble Dectin-fusion proteins were purified by affinity chromatography using a protein G column (GE Healthcare, Little Chalfont, United Kingdom). For ELISA-based binding assay, test antigens were coated in a concentration of 20 µg/ml in PBS on medium-binding half area 96-well microtiter plates (Greiner Bio-One, Kremsmünster, Austria) at 4 °C overnight. Wells were washed with 0.05% Tween 20 in PBS and blocked with 1% BSA in PBS at RT for 2 h. After further washing, Dectin-1-Fc and Dectin-2-Fc were added to respective wells at a concentration of 8,4 µg/ml in lectin buffer (50 mM HEPES, 5 mM MgCl2, 5 mM CaCl2, pH 7.4) at RT for 1 h, followed by incubation with peroxidase-conjugated anti-hFc antibody (Dianova, Hamburg, Germany) 1:5000 in reagent diluent (1% BSA, 0.05% Tween 20 in PBS) at RT for 1 h. Finally, colorimetric detection was performed using o-phenylenediamine dihydrochloride as substrate and subsequent measurement at 495 nm in a Multiskan GO spectrophotometer (Thermo Scientific, Waltham, Massachusetts, USA).

Statistics: Cytokine expression in response to different stimuli was analyzed by a one-way repeated measures F test (Friedman-test) with Dunns post-testing in order to correct for repeated measures. To calculate differences between receptor- or kinase-blocking and respective controls, non-parametric paired testing (Wilcoxon-matched pairs test) was applied due to non-gaussian distribution of data. Internalization of ligands compared to the negative control was analyzed by Wilcoxon-matched pairs test as well. Binding of candidate ligands to Dectin-1 was analyzed using a one-way ANOVA with Bonferroni post-testing. (GraphPad Prism 5; GraphPad Software, San Diego, CA). Differences were considered significant if *p* < 0.05.

The datasets generated during and/or analyzed during the current study are available from the corresponding author on reasonable request.

## Supplementary information

supplementary info
