# Supplementary material for: Human thioredoxin, a damage-associated molecular pattern and Malassezia-crossreactive autoallergen, modulates immune responses via the C-type lectin receptors Dectin-1 and Dectin-2
Source: Sci Rep. 2019 Aug 1;9:11210. doi: 10.1038/s41598-019-47769-2 (PMC6671947; doi:10.1038/s41598-019-47769-2)
Supplement: Supplementary file 1 — supplementary info [file 41598_2019_47769_MOESM1_ESM.pdf]

Human thioredoxin, a damage-associated molecular pattern and *Malassezia*-crossreactive autoallergen, modulates immune responses via the C-type lectin receptors Dectin-1 and Dectin-2

Roesner, L.M.,[1*$] Ernst, M.,[1$] Chen, W.,[1] Begemann, G.,[1] Kienlin, P.,[1] Raulf, M.K.,[2,3] Lepenies, B.,[2] and Werfel, T.[1]

Supplementary Information

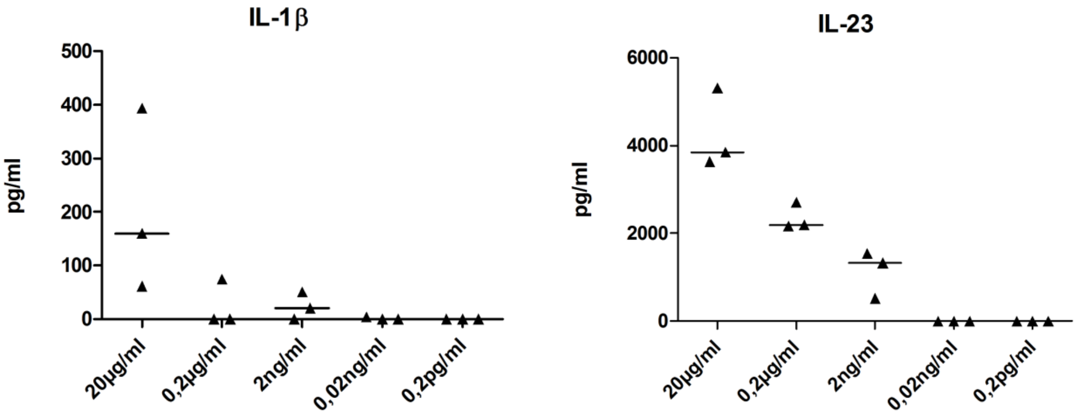

Figure S1. Cytokine release from moDC after application of LPS. In the experimental setup applied in this work, LPS contaminations of 2ng/ml or higher would have had the potential to lead to false positive effects.

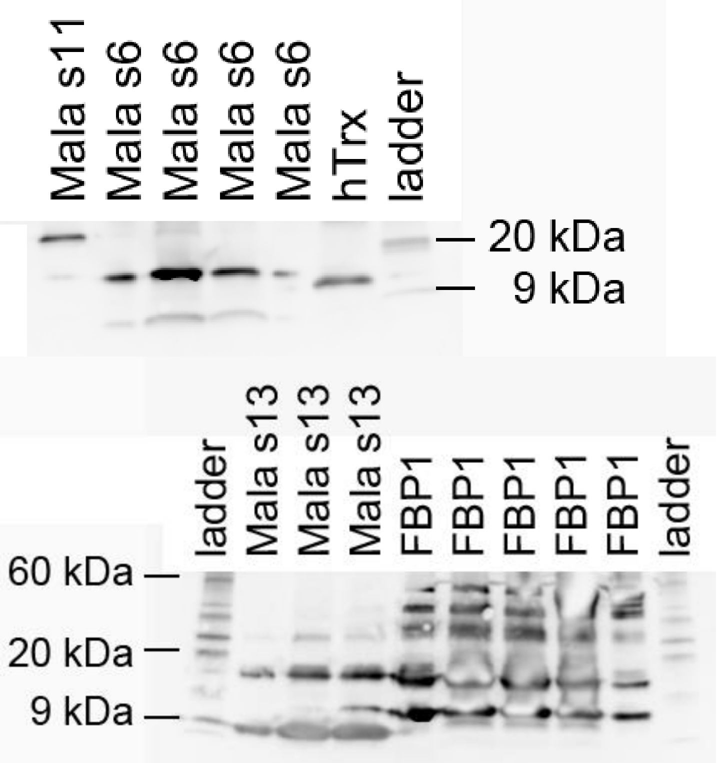

Figure S2. Detection of recombinant proteins applied to the Dectin-1 binding platform. Recombinantly expressed, 6xHis-tagged allergens of *Malassezia*, Mala s6, s11, and s13, as well as its human paralogue thioredoxin (hTrx) and the fibronection binding protein 1 (FBP1) from *Staphylococcus aureus* were detected via an antibody specific to 6xHis-tag.
